# Ultra-Wideband Indoor Positioning and IMU-Based Activity Recognition for Ice Hockey Analytics

**DOI:** 10.3390/s21144650

**Published:** 2021-07-07

**Authors:** Robbe Vleugels, Ben Van Herbruggen, Jaron Fontaine, Eli De Poorter

**Affiliations:** IDLab, Department of Information Technology, Ghent University-imec, 9000 Ghent, Belgium; robbe.vleugels@ugent.be (R.V.); jaron.fontaine@ugent.be (J.F.); eli.depoorter@ugent.be (E.D.P.)

**Keywords:** ultra-wideband (UWB), indoor localization, ice hockey, activity recognition, IMU, machine learning, convolutional neural network (CNN), carbon fiber composite, wearable

## Abstract

Currently, gathering statistics and information for ice hockey training purposes mostly happens by hand, whereas the automated systems that do exist are expensive and difficult to set up. To remedy this, in this paper, we propose and analyse a wearable system that combines player localisation and activity classification to automatically gather information. A stick-worn inertial measurement unit was used to capture acceleration and rotation data from six ice hockey activities. A convolutional neural network was able to distinguish the six activities from an unseen player with a 76% accuracy at a sample frequency of 100 Hz. Using unseen data from players used to train the model, a 99% accuracy was reached. With a peak detection algorithm, activities could be automatically detected and extracted from a complete measurement for classification. Additionally, the feasibility of a time difference of arrival based ultra-wideband system operating at a 25 Hz update rate was determined. We concluded that the system, when the data were filtered and smoothed, provided acceptable accuracy for use in ice hockey. Combining both, it was possible to gather useful information about a wide range of interesting performance measures. This shows that our proposed system is a suitable solution for the analysis of ice hockey.

## 1. Introduction

Ice hockey is sometimes called the fastest team sport in the world. Ice hockey players require a broad range of skills, such as power, endurance, good balance and fast reaction times, as described in [1]. These skills are all basic elements constituting to the sport specific techniques, such as skating, stick/puck handling and body checking. Players can reach speeds of up to 40 km/h while skating. They also have to keep track of the puck, a small disk made out of vulcanized rubber, which can reach speeds of 170 km/h. Similar as for other sports, in ice hockey objective data can support coaches in giving feedback to players and analysing games.

Currently, useful information is mainly registered manually by a coach or an assistant, especially in a non-professional context. An easy to operate but accurate system would be a useful addition; capturing and analysing data could then be performed at any time. Research towards the application of technology in sports analysis has been a hot topic in the past couple of years.

Study subjects include indoor localisation, activity recognition, injury prevention, health monitoring and referee assistance with camera-based systems, such as goal line technology. In this paper, we focus on sport analytics by means of localization and activity recognition. The primary goal is to assess the possibility of using ultra-wideband (UWB) in ice hockey for localisation together with activity detection and recognition of ice hockey activities.

Several indoor positioning technologies have been investigated recently [2], such as WiFi, Bluetooth, Radio Frequency Identification and Zigbee, but UWB is one of the the most promising. Ultra-wideband is a wireless technology that uses radio frequency pulses with a very short time duration (picoseconds to nanoseconds) to transmit information. Short pulses result in a large bandwidth and, therefore, spread the energy of the signal over a large frequency band. Advantageous properties of UWB include its excellent robustness against shadowing, good multipath resolution, large channel capacity (high data rates) and low energy consumption [3]. This makes it such that centimeter-level accuracy can be obtained [4]; therefore, UWB technology is quickly becoming the dominant technology in the indoor positioning market.

Human activity recognition has been an active research topic in recent years due to its broad range of possible applications. The increase in popularity is mainly due to two reasons. First of all there is the increased usage and availability of smart watches and other wearable devices. With the recent advancements in technology and miniaturization, these are often equipped with the necessary sensors [5], such as micro-electromechanical systems (MEMS) [6], and are relatively cheap. Secondly, recent advancements in computer vision, machine learning, artificial intelligence and the Internet of Things (IoT) have made it possible to obtain good results that are practically applicable [7].

In this paper, we use a combination of the previously discussed technologies to obtain relevant information about ice hockey players. UWB technology is used to determine the position of players on the ice during a game or practice. From this information, useful characteristics can be derived, such as the covered distance and reached speeds. Additionally, an Inertial Measurement Unit (IMU) is attached on the stick to obtain data about shots and other activities, and activity recognition can then be applied on this data. Both are combined to create a broader picture of what a player is doing at a certain time; a process that is more generally called sensor fusion. This information is useful for both coaches and players to monitor and improve their game. The main innovations of this paper are:

- We detect and classify six ice hockey activities using a single stick-mounted IMU.
- We use ultra-wideband for the localisation of ice hockey players on an ice rink.
- We investigate the influence of carbon fibre composite ice hockey sticks and body presence on the UWB positioning.
- We combine localisation and activity recognition for ice hockey analytics.
- The used data set has been made open source (Available at: gitlab.ilabt.imec.be/bvherbru/dataset-uwb-imu-ice-hockey (accessed on 11 June 2021 )).

The remainder of this paper is structured as follows. Section 2 describes the related work for UWB athlete tracking and IMU-based activity recognition. Section 3 discusses the classification approach in order to classify six ice hockey specific activities, including data collection, processing and the used model. Section 4 is dedicated to the evaluation of UWB for ice hockey, determining the influence of the materials on signal propagation and using a 25 Hz Time Difference of Arrival (TDoA)-based UWB system for positioning on the ice. In Section 5, both are combined such that a broader analysis can be made. Finally, in Section 6, a conclusion is drawn based on the discussed results.

## 2. Related Work

Ultra-wideband localisation and human activity recognition have been actively researched in recent years. Not only for general sport movements [8,9] but also for specific sports, such as table tennis [10], baseball [11], basketball [12], volleyball [13] and swimming [14].

### 2.1. Usage of Ultra-Wideband in Sport Analytics

In [15], an UWB positioning system was used to track athletes during indoor wheelchair court sports. Six anchors are used to cover an indoor wheelchair rugby court (28 m × 15 m). They concluded that athlete tracking could be achieved with an average error of ±0.37 m (σ = ±0.24 m) indoor. The total distance covered can be computed after data processing. In [16], the validity of a commercial 10/20 Hz UWB local positioning system was assessed for handball-specific movements indoor. They concluded that it had acceptable performance, however, that care should be taken when monitoring accelerations and decelerations especially at the sides of the field.

This observation was made in other publications as well [17]. In [18], an UWB real time location system was used to track athletes in two large-scale scenarios, once for indoor track cycling and once on an indoor running track. Using several localisation algorithms, an average error of about 20–30 cm was obtained. The authors in [19] studied the application of UWB localization in order to determine the position of a tennis player within the tennis court at the moment when the racquet hits the ball. An IMU was used to detect this impact, and time synchronization was applied to sync both data streams. The obtained mean squared positioning error of the player’s trajectory was 14.5 cm, and, for the mean squared positioning error of the player’s position at impact, a value of 7.4 cm was obtained.

There is, to the best of our knowledge, only one paper reporting UWB localisation in ice hockey [20]. They measured the average skating distances, average time on ice and reached velocities of players at 10 Hz with 24 anchors installed inside a stadium. The data were used to make conclusions about positional differences in the distance and intensity of skating during the course of a match. It is, however, uncertain how accurate UWB is in order to determine the position of ice hockey players since they assumed the commercial UWB system to be accurate enough for the analysis of indoor team sports.

### 2.2. Activity Detection and Recognition in Sports

In [21], a general system is proposed for the analysis of swing-based sports. Combined over three sports (tennis, badminton and squash), they were able to detect shots with a 92% accuracy and classify shots with a 87% accuracy. In [22], an approach was proposed to successfully classify badminton strokes using a convolutional neural network and a deep neural network with a precision of 99%. In [23], a wrist worn IMU was used for shot detection and classification in tennis. Shot detection was achieved with a 99% accuracy and classification with a 90% accuracy using quaternion-based dynamic time warping.

Only [24] has attempted something similar in ice hockey, a sensorized hockey stick was created to perform research on shooting techniques in ice hockey. A stick was equipped with motion sensors, strain gauges and pressure-sensitive potentiometers. The motion sensors were positioned in a case that fit in the upper end cavity of the stick. The strain gauges measured the flexion of the stick and were attached at the outside of the stick together with the pressure sensitive potentiometers reading out the hand positions of the player.

This configuration is not suitable for in game usage, as multiple sensors are applied to the outside of the stick. In our paper, no hand positioning and stick flexion data are available. Additionally, we distinguish between all four possible ice hockey shots instead of the two used in [24]. An accuracy of 92.9% was achieved in their case.

In Table 1, we provide a short overview of the previously conducted, related research. In contrast to most of the publications, we combine both activity recognition and positioning. Ice hockey is only found as a subject in a minority of papers. Additionally, tags and sensors are mostly worn on body, which is not ideal for performance. The human body has a significant impact on signal propagation [25,26], and body worn sensors are also less comfortable for players. Placing a tag combined with an IMU inside of a stick is a new application that will be further investigated in this paper.

## 3. Activity Classification

There are different ice hockey activities. In this paper, the four possible shots (i.e., slap shot, snap shot, wrist shot and backhand shot), stick handling and the hockey stance were considered.

### 3.1. Possible Activities

Whenever a quick shot on the net is necessary, a wrist or snap shot is executed. The snap shot is a similar but a shorter version of the wrist shot in which the puck is not guided and the player immediately pushes his stick into the ice without making contact with the puck while shooting.

If there is more time, a slap shot can be performed, which allows the player to put more power into the shot such that the puck can reach higher velocities. This shot is, however, also more predictable for the goalie and less accurate [27]. A unique characteristic of the slap shot is the backswing in which the stick is brought up in the air behind the player, building up potential energy before swinging it down to the ice, shooting the puck. In the wrist shot, on the other hand, the puck is always making contact with the stick blade. The player guides the puck towards his back leg before shooting.

The final type of shot is the backhand, which is performed when the puck is on the backhand side of a player. This shot can be performed in several manners; however, players will typically lean on their front leg to put weight and, thus, power into the shot. The puck will roll from the stick heel to the stick toe during the shooting motion. It is important to note that there are both right and left sided hockey sticks. Therefore, a shot from the left side can be either a forehand or a backhand shot, depending on the player.

**Figure 1:**
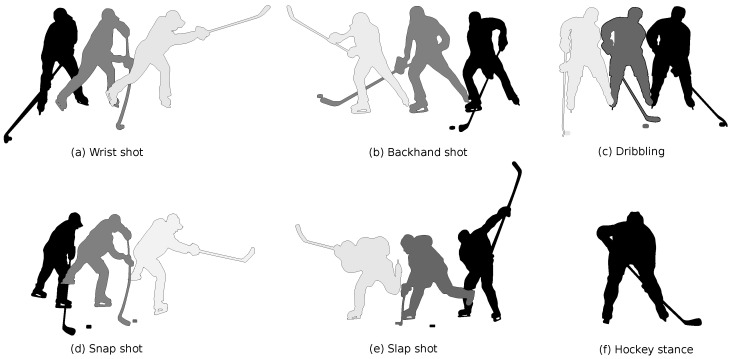
Illustration of the six activities, the correct sequence of movements is indicated by the transition from darker to lighter colours.

The fifth activity is puck handling, more specifically dribbling. This movement allows the player to deke and cover the puck in game situations. The puck is continuously moved from forehand to backhand and vice versa in a fluent motion. The final activity is a hockey stance, the player keeps his back straight, head up, chest out and knees bent. This position is used at a face-off or whenever a player is ready to receive a pass. All activities are illustrated in Figure 1, indicating the correct sequence of movements by the transition from darker to lighter colours.

### 3.2. Data Collection for Activity Recognition

The first data collection campaign (out of four), was performed at an indoor ice rink (Ice Skating Center Mechelen, Belgium [28]) with five participants. Each participant wore two tri-axial accelerometer and tri-axial gyroscope-based IMU’s. All participants were male ice hockey players playing in the BeNe-league, the best ice hockey competition in Belgium and the Netherlands. Using five players, both left and right sided, ensured that the input was as varied as possible since the execution of a shot is different for each player. An overview of all collected data campaigns is given in Table 2.

#### 3.2.1. Used Hardware

The used IMU was an off-the-shelf Axivity AX6 6-Axis Logging Device [29]. The key features are its small form factor 23 × 32.5 × 8.9 mm, low weight (11 g), built in 1024 Mb flash memory and 16 bit resolution. This device has two MEMS [6] sensors on the inside with the ability to record data: an accelerometer to detect linear acceleration changes and a gyroscope measuring angular velocity (rotation). Both sensors have three measurement axis, i.e., *x*, *y* and *z*. The design was purposely made similar to the AX3 for maximum compatibility. The AX3 was previously successfully used to recognise certain physical training exercises [30]. Due to its small weight and form factor, the AX6 is perfect to attach on a body or stick with minimal disturbance to the player.

#### 3.2.2. Measurement Setup

**Figure 2:**
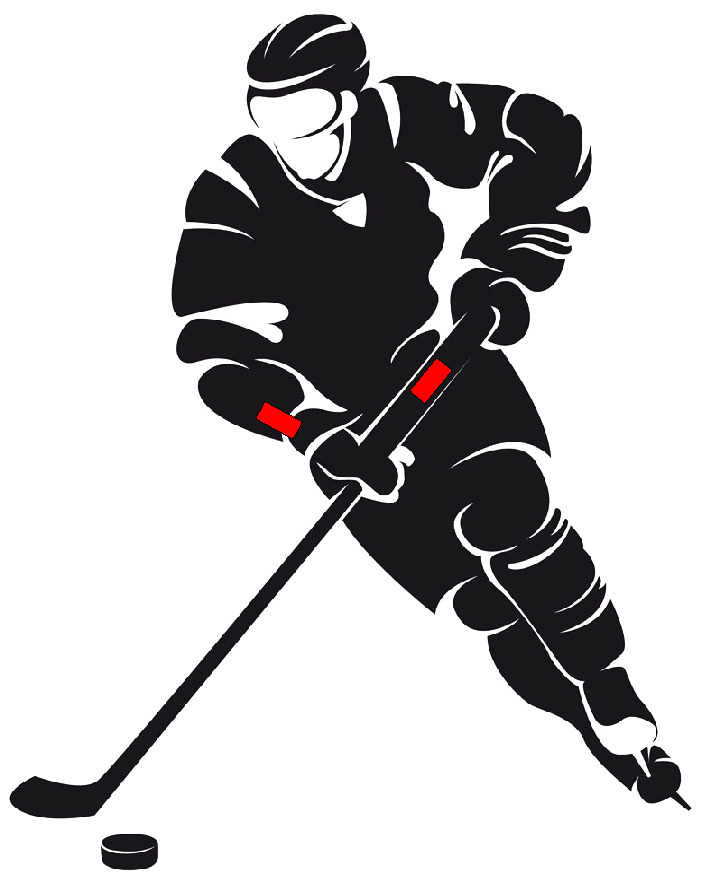
Positioning of the sensors on a hockey player.

Each participant was asked to perform the six activities multiple times, resulting in the recorded dataset consisting out of 1,724,280 data points. The data were recorded at a sample frequency of 400 Hz, and, as such, the recorded data were approximately 72 min long. For each of the data points, six values are available representing the six axes of the used IMU: acceleration in the *x*, *y* and *z* directions and rotation around the *x*, *y* and *z*-axes. One AX6 device was attached to the stick of the player and a second one to the wrist of the shooting side as illustrated in Figure 2. The data from the wrist worn IMU is currently not used. The accelerometer signal had a range of ±16 g and the gyroscope had a range of ±2000 dps.

To process the raw sensor output data, the AX3/AX6 OMGUI Configuration and Analysis Tool was used. This tool can convert the raw data to a .CSV format, which is easy to use in further processing with Python and the pandas library [31]. All activities were manually extracted out of the complete measurements and labelled. Based on the data, a duration of 1.5 s was chosen as the length of an activity. The result was a total of 413 available activities, and a summary for each of the players is given in Table 3.

### 3.3. Classification

#### 3.3.1. Data Preprocessing

The rather limited dataset was expanded by means of data augmentation: left sided players were converted to right sided players and vice versa. Due to the used sensor orientation, the Ay, Az, Gx and Gy axes were inverted to achieve the desired conversion. This resulted in a dataset with both left and right sided players such that the model could distinguish activities from both. In our case, the total amount of data was, therefore, doubled. Several data preprocessing techniques were applied.

As proposed by [22], the magnitudes also possess information for the model to learn in addition to the patterns in the signal. Hence, the signal was scaled, and no normalisation techniques were applied. Afterwards the data were downsampled to lower the complexity, memory footprint and power consumption of the model. A sampling rate of 100 Hz yielded good results and is used further on. With this sampling frequency, the input had 150 samples of six measured features (Ax, Ay, Az, Gx, Gy and Gz) for each activity. As a final preprocessing step, the data were divided into three data sets. The training set, validation set and test set.

#### 3.3.2. Convolutional Neural Network (CNN)

To allow activity classification, a convolutional neural network [32] was constructed using the Keras library in Python. The model structure was chosen to be sequential such that it consisted out of a linear stack of layers. The first part of the model contains several convolutional and pooling layers in order to perform feature extraction. The first layer is a 2-dimensional convolutional layer in which 32 (3 × 1) kernels are used to shift over the input data while performing multiplications, resulting in 32 feature maps.

The activation function is a Rectified Linear Unit (ReLU), and an L2 kernel and a bias regularizer were applied, both with a value of 0.01. These regularizers apply a penalty to the weights and the bias of the model in order to prevent overfitting. In the L2-regularizer, a certain percentage of the sum of the squared weights was used as a penalty to the loss function.

The second layer is a maxpooling layer in which a (2 × 1) kernel is used to shift over the feature maps. They were downsampled by a factor of two. At every stride, the maximum value in the kernel window is preserved while the other value is discarded. The third layer is a similar, but smaller, 16 kernel 2D-convolutional layer, and it is succeeded by a final 16 kernel convolutional layer. The resulting data were then flattened into a 1-dimensional array as input for the second part of the model, which consists of several fully connected dense layers. Each node is directly connected to every node in both the previous and the next layer.

The first fully connected layer consisted out of 64 nodes, and the same kernel and bias regularizer are applied with the ReLU activation function. This is succeeded by a 35% dropout layer. Dropout layers are another generalization technique in which a random number of layer outputs are randomly ignored with a certain probability. This succession of layers is repeated once but with a smaller amount of nodes and a different dropout percentage. The final layer is a six-node dense layer since we are trying to classify six activities. In this layer, the activation function is softmax, the result can then be interpreted as a probability because the output sums up to one. In total, the model consists of 291,926 trainable parameters. Its structure is summarised in Table 4.

The used optimizer is adam, a stochastic gradient descent method that is based on the adaptive learning rates of parameters. Adam performs smaller adaptations of the learning rate for parameters associated with frequently occurring features, and larger updates (i.e., high learning rates) for parameters associated with infrequent features. In order to adapt the global learning rate, a learning rate reduction of 10% was applied. Whenever the accuracy is not improving during a period of 20 epochs, it is assumed to have reached a plateau. The global learning rate is then reduced.

#### 3.3.3. k-Fold Cross Validation

In order to have an assessment of the generalisation ability of the model, k-fold cross validation was used. k-Fold cross validation is a data resampling method in which the available data set is randomly partitioned into *k* disjoint and approximately equal sized sets. The model is trained k-times, each time using (k−1) folds as the training set and one fold as the test set. As such, every subset is used once as a test set. The performance of the model is measured by averaging the performance measurements on the *k* validation sets [33]. Five-fold cross-validation was applied in this paper (k=5). For each iteration, a different, unseen player (both L and R) was used as the test fold.

#### 3.3.4. Machine Learning Hardware

The machine learning model was trained using Google Colab with GPU hardware acceleration enabled. Google Colab is a cloud service from Google in which Python code can be easily written and executed in the format of a Jupyter notebook, without any setup. This is very popular for machine learning purposes since it supports most popular machine learning libraries, hence, our choice to use it. In [34], it was shown that Colaboratory hardware resources reached a performance similar to dedicated hardware.

The used CPU is an Intel Xeon (exact model not further specified) with a clock frequency of 2.3 GHz, the GPU is a Tesla K80 from Nvidia. At a 100 Hz sampling frequency, one fold, 200 epochs, has a duration of 95 s. When GPU acceleration is disabled, each fold has a duration of 200 s. This means that, for the complete k-fold cross validation, GPU acceleration provides a significant training time decrease (of 52%) from 17 to 8 min.

### 3.4. Results

**Figure 3:**
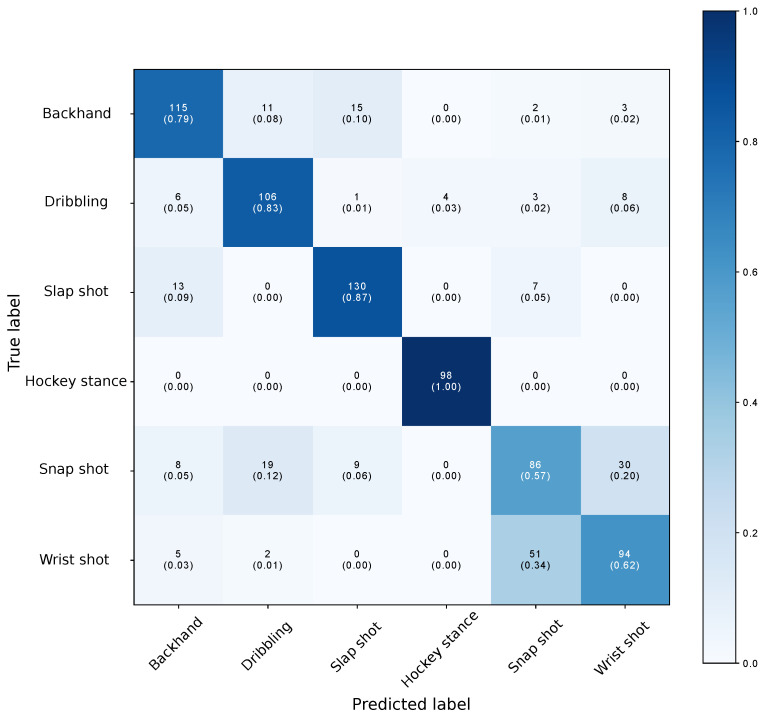
Confusion matrix for k-fold cross validation with six activities on unseen players. There is clearly confusion between the similar wrist and snap shots.

The confusion matrix aggregating the results from the k-fold cross validation is given in Figure 3. The accuracy for each of the players is given in Table 5, with accuracy defined as:

$$\mathrm{accuracy}=\frac{\mathrm{TP}\;+\;\mathrm{TN}}{\mathrm{TP}+\mathrm{TN}+\mathrm{FP}+\mathrm{FN}}$$

TP stands for True Positive, FN for False Negative, FP for False Positive and TN for True Negative. The model had an averaged accuracy of 76% but accuracy varied greatly between players with a minimum value of 69% and a maximum value of 90%. This indicates that players did not perform activities exactly the same, and we believe that a larger data set covering more variations would, therefore, improve the result. The model is clearly uncertain between snap shots and wrist shots, which is confirmed by looking at the output probabilities.

These are for both activities larger than 0.4, an observation that can be explained by the fact that the wrist and snap shot are very similar to each other. During a game situation, most players actually perform a shot that is in between the two. Hence, the model was also evaluated aggregating the two shots into one label. The total accuracy increased to 87%, and the results also varied from player to player.

Another option is to use some form of calibration or continual learning. In this case, the model was trained using data from all players and tested using a test set of unseen data from the same players. This increased the model accuracy to 99%, both for the model with and without combining snap and wrist shots. The models were evaluated using 10-fold cross validation on randomly generated folds.

### 3.5. Detection and Recognition

A sliding window with a 16.67% overlap was used to detect peaks corresponding to activities in the IMU data. Based on the values of acceleration and rotation, we determined whether there was a significant peak present in the window. In that case, an integration was performed over a smaller window for which the position was determined based on the moment of maximal rotation. This prevented every momentary peak in one of the considered values being registered as an activity.

**Figure 4:**
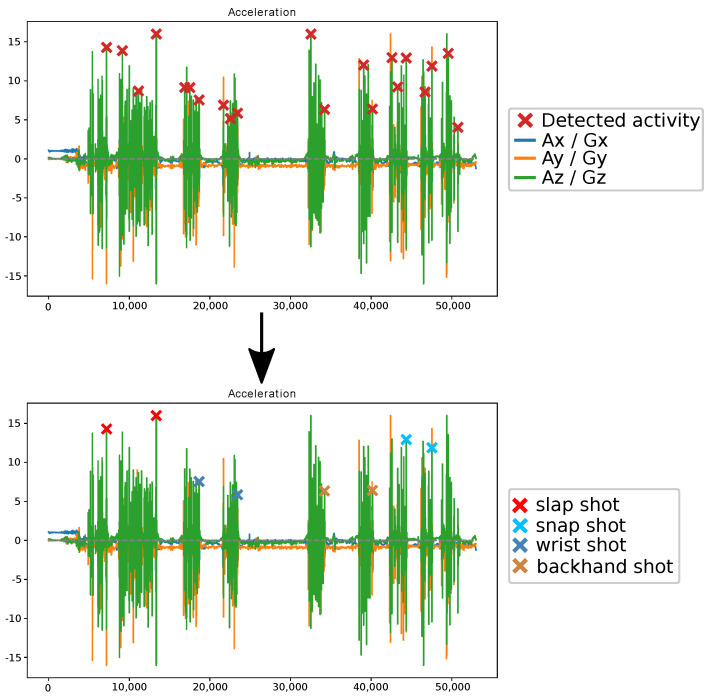
Process of detecting and recognising activities from the IMU data of repetition 1.

If the calculated integral was larger than a certain threshold, the peak was considered as an activity. It could then be processed as discussed in Section 3.3.1 to give it to the machine learning model for classification. Based on the result, the peak is either one of the shots or a dribble. Dribble peaks were discarded, and peaks from shots are indicated with a colour corresponding to the type of shot recognised. An example of the process is given in Figure 4, where a player was asked to perform each type of shot two times while dribbling and skating.

In order of appearance, two slap shots, wrist shots, backhand shots and snap shots were executed. These were correctly detected and recognised. This experiment was repeated three times, 23 of the, in total, 24 shots were correctly detected and recognised. For the other activities, corresponding to dribbles, one dribble was wrongly classified as a shot. Hence, in total, two activities were incorrectly predicted during the course of the three repetitions. The results for these three repetitions are summarised in Table 6.

## 4. Ultra-Wideband Positioning in Ice Hockey

To enable position information during ice hockey, an UWB system was used. A time difference of arrival based approach was chosen due to the good scalability to multiple players, low power consumption for tag nodes and the possible high update rate compared to TWR positioning systems. For localisation with TDoA, a tag and at least three anchors need to be available to have a 2D position estimate. The tag is small such that it has limited disturbance for the players. It is equipped with a DWM1001 from Decawave, which has shown better performance compared to other commercially available UWB systems [35]. It has a monopole UWB PCB trace antenna, a sub-GHz antenna and is small enough that it can fit inside of a hockey stick.

**Figure 5:**
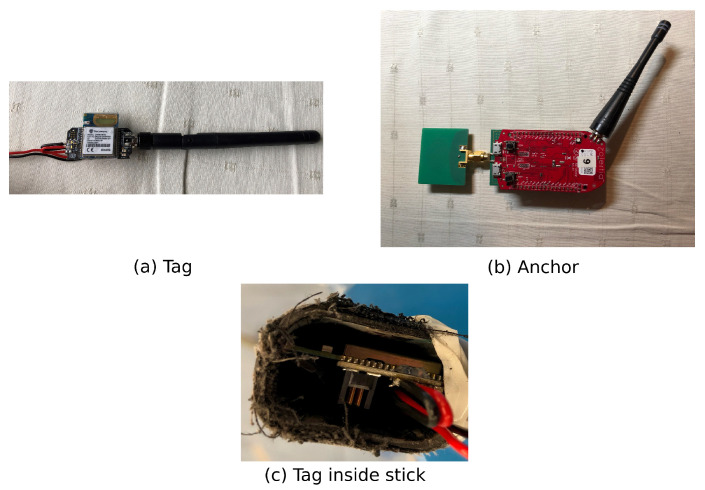
The used tag and anchor hardware; a tag could be inserted inside a stick.

The Wi-Pos system [36] was used for the anchors; it consists of the Zolertia RE-Mote Internet of Things platform together with a custom UWB shield. It is based on the same DW1000 transceiver but uses an external UWB antenna connected to the board via a SMA connector. It also features a sub-GHz backbone that can be used for long range, reliable and power efficient communication. Both are depicted in Figure 5.

### 4.1. Material Influence on UWB Signal Propagation

Hockey sticks are made out of state-of-the-art one-piece composite materials, mostly carbon-fibre-based [37]. These materials are very strong, and therefore sticks can be hollow to reduce weight. This is useful to position tags on the inside of the stick, reducing the disturbance of the player’s movement by the hardware. Therefore, the influence of the stick’s material was investigated together with the additional influence of a nearby body and hands. In order to determine the influence of the stick’s material on UWB signal propagation, a second measurement campaign was organised. A measurement was performed with one tag and one anchor in two situations.

In the first situation, a tag was used in free space, meaning it was not inserted in the stick and was used in an open area. In a second test, this same tag was placed inside of the stick, and a measurement was performed on the same location, as indicated by a measuring tape for reference. Measurements were performed at fixed distances from the anchor. The obtained data points, for distances at which both situations could still be received, are given in Table 7.

A first observation from the measurement was the influence on the maximum ranging distance. The maximum distance for a tag inserted inside of the stick was equal to 20 m, while the maximum distance for a tag in free space was equal to at least 35 m. No further measurements were performed beyond this distance. This indicates that the stick’s material resulted in signal attenuation, decreasing the range significantly.

A second observation was the influence on the distance estimate between the anchor and tag. When the tag was inserted inside of the stick, the estimated distance differed with a value of 43 mm on average, when compared to a tag outside of the stick. The permittivity of the stick’s material resulted in an estimated distance that was slightly larger than in free space. The real part of the permittivity was estimated to be 4.3, a value corresponding to those for similar carbon-fibre composites found in [38,39,40].

(1) $$\begin{array}{c} \epsilon_{real}\approx {\left(\frac{c}{v_{stick}}\right)}^{2}={\left(\frac{c}{\frac{▵x_{stick}}{▵t_{stick}}}\right)}^{2}=\;4.3 \end{array}$$

with *c* as the speed of light, $▵x_{stick}$ as the average difference in the reported distance and $▵t_{stick}$ as the time needed to propagate through the stick’s material as derived based on the measurement. The influence of the stick on the position estimate is discussed further on.

### 4.2. Body and Material Influence on the Position Accuracy

To determine the additional influence of a body in a more realistic situation, another measurement was performed in an indoor environment at the IIoT lab in Ghent. There has already been research towards the human body impact on UWB radiation [25,26,41,42,43] and for the best body placement of a tag [18,44].

The question remains regarding what the influence would be in several ice-hockey-related situations. The most interesting and realistic situation is a tag inserted inside of a stick with a body and hands near it. In this setup, eight anchors were positioned around the room, and one tag was used in four possible situations. The stick and the location of the tag were tracked with a MOCAP system as the ground truth. In the first situation, the tag was attached to the outside of the stick. In the second situation, the tag was inserted inside the stick without anyone in the surroundings. In the third situation, the tag was inserted inside the stick, and a body was positioned near the stick in between two of the eight anchors. In situation four, the tag remained in the same position but a hand was wrapped around the stick at its location.

**Figure 6:**
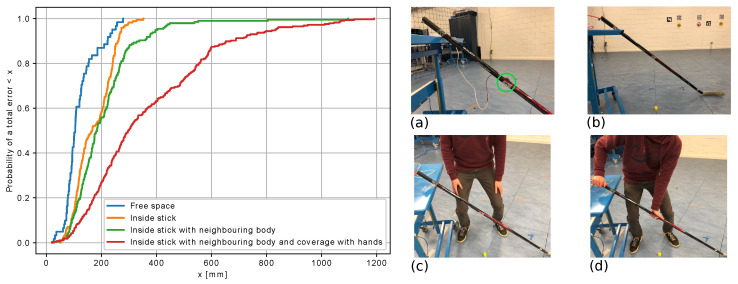
Cumulative distribution functions of the positioning error for a tag in (**a**) free space, (**b**) inside a stick, (**c**) inside a stick with a nearby body and (**d**) inside a stick with hands surrounding. The tag was always positioned at the location of the green indication.

The cumulative distribution functions of the positioning error were determined by comparing the MOCAP position with the derived position estimate from the UWB for each of the situations. The results are given in Figure 6 together with an illustration of the four situations. The first three situations had small positioning errors with a 50th percentile smaller than 20 cm and a 90th percentile smaller than 35 cm. When hands were surrounding the tag, the performance was worse. The 50th percentile positioning error was 30 cm, and the 90% positioning error was 66.2 cm.

This was more than double the error of the other cases. In summary, inserting the tag inside a hockey stick did not have a significant influence on the accuracy of the position estimate, as long as the tag was positioned such that it was not covered by hands. There was, however, a significant signal attenuation that reduced the range.

### 4.3. Player Localisation on the Ice

A 25 Hz time difference of arrival based UWB system [36] was used to analyse the movements of players on the ice. As mentioned earlier, time difference of arrival has the benefits of low power consumption and excellent scalability compared to other techniques, such as two-way ranging. All timestamps were collected at the anchor nodes and combined at the edge node. The edge node calculated the time differences between every anchor pair and pairs with successful UWB reception and calculated the position with a least squares approach.

#### 4.3.1. Ice Rink Set Up

Nine anchors were installed on a surface area of 65.25 × 32.5 m, and the ice surface itself had a size of 60 × 28 m. Each anchor was connected to a Raspberry Pi, which stored the data logged by the anchor and could be controlled from a central laptop using SSH. The process of activating and deactivating the logging was automated using a batch script, and the positions were calculated in post-processing. Athletes wore a back mounted tag against the body, and the data were processed using Python.

**Figure 7:**
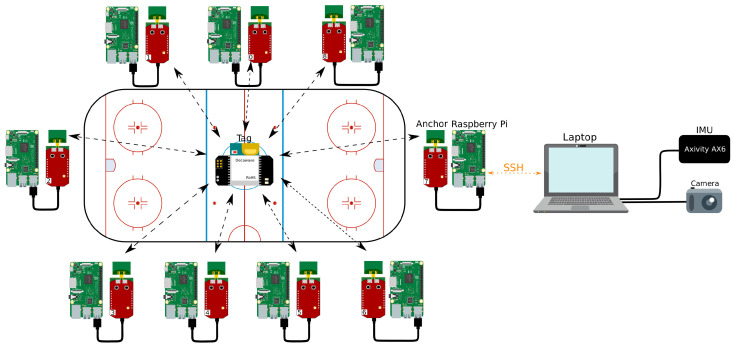
The complete ice rink setup for the UWB system. Nine anchors were mounted around the ice surface using TDoA at 25 Hz.

The complete setup is illustrated in Figure 7, including a camera and IMU. The video footage from the camera was not calibrated and could not provide accurate ground truth information. Therefore, it was used as a visual comparison of the derived position. The IMU was used to measure activity data during the UWB measurements later on when combining both.

#### 4.3.2. Data Collection for UWB Positioning

A third measurement campaign, designed for UWB evaluation at the ice rink, was performed. First, the accuracy was determined at several fixed, known locations with a tag mounted on a tripod. In these stationary situations, the system had an error smaller than 1 m, which is accurate enough for applications in ice hockey. As a dynamic measurement, a player was asked to skate around the five indicated face-off circles at three different speeds: slow, moderate and fast skating.

#### 4.3.3. Results

**Figure 8:**
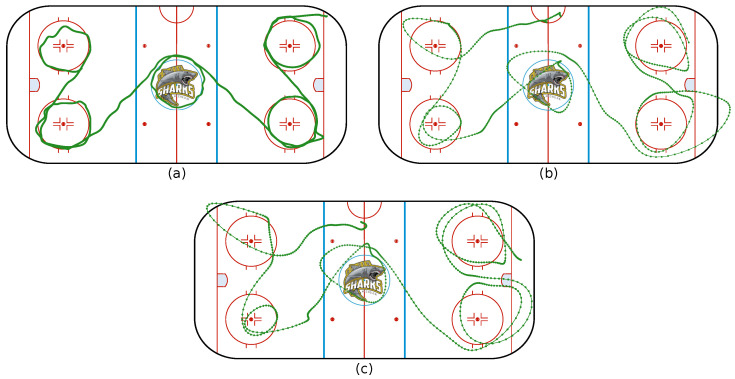
Localisation of a player skating around the five face-off circles at (**a**) low speed, (**b**) average speed and (**c**) high speed.

First, outliers were removed from the raw data. Outliers were determined based on the cumulative distribution function of the difference in distance between consecutive points, and the 90th percentile was chosen as a threshold. Points above this threshold were considered as outliers and removed. After the removal of outliers, a Kolmogorov–Zurbenko filter was applied to smooth the data. The results of the measurement, after processing, are shown in Figure 8.

It can be seen that, comparing the derived trajectory with the fixed face-off circle positions, the position estimate was good for low speeds while becoming less accurate for higher speeds. Other measurements also indicated that positioning was sufficiently accurate for certain applications and speeds but inadequate for others. High speeds, sudden stops and accelerations were the most difficult to measure. Although the accuracy degraded for faster scenarios, the UWB localization system allowed for performance analysis on the general movements, the time on ice and the tactical behaviour of a player.

Further improvements could be obtained using a similar system to [20]; the accuracy of their system was, however, not assessed. They used a 10 Hz commercial system with 24 anchors to cover a similar surface area and a combination of ranging techniques (TDoA, TWR and AoA) in order to determine the position. We only used TDoA with nine anchors but at a higher update rate of 25 Hz.

## 5. Combining Activity Classification and Player Localisation

Using the UWB and activity recognition, a wide variety of characteristics can be determined. We focused on the application of both in order to count the amount of shots made by a player, which shots and from which location, since this is valuable tactical information for both players and trainers. In this fourth measurement campaign, a player was asked to perform each type of shot two times in a single exercise while skating and dribbling. The first two shots, both slap shots, were executed around the blue line of the upper right quadrant.

**Figure 9:**
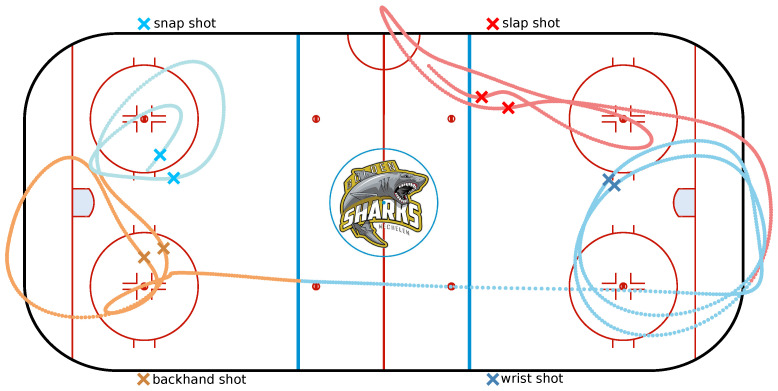
Indicated locations of the eight shots on the determined trajectory after application of a Kolmogorov–Zurbenko filter.

Secondly, two wrist shots were made from the slot in front of the right goal. Third, two backhand shots were performed from the slot in front of the left goal. Lastly, two snap shots were made from the upper left face-off circle. The activity detection and recognition for this experiment were already discussed in Figure 4. It can be seen from Figure 9 that the locations of the shots are correctly indicated. Combing the activity classification and localisation, therefore, makes sense.

The availability of both enables many possible ice hockey performance measures to be tracked. An overview of these possible ice hockey performance measures is given in the first column of Table 8. In the second and third column, the necessary input and relevance for strategy and single player feedback are given. The relevance score is given on a scale from one to ten, a higher number means that a measurement is more relevant. In the last column, it is indicated whether the measurement was possible using the proposed system.

## 6. Conclusions

In this paper, we presented a system to evaluate the performance of ice hockey players based on both ultra-wideband localisation and IMU-based activity recognition. Up to now, statistic information has been collected manually, showing the need for an easy to deploy analytic system. We showed that data captured by a stick-worn IMU from six ice hockey activities could be distinguished by a CNN at a sampling frequency of 100 Hz. For completely unseen players, a classification accuracy of 76% was obtained. When adding player specific data for training, this increased to a maximal accuracy of 99%.

Activities were extracted and classified out of a dynamic skating scenario using a peak detection algorithm with a sliding window. We also validated that, when filtered and smoothed, a nine anchor 25 Hz TDoA-based UWB system provided acceptable validity for use in ice hockey. Tags can be inserted inside sticks, and therefore the influence of the stick’s material was investigated together with the additional influence of a nearby body and hands. The stick had a negligible influence on the position estimate but resulted, however, in a large signal attenuation, decreasing the range significantly.

The presence of a body and hands decreased the accuracy and could block the line of sight to multiple anchors, which also impacted the range. Both technologies can be combined to gather useful information about a wide range of performance measures. One option is to determine which player performed a certain sort of activity, such as shots, at a certain time and location. The discussed technologies and methods are, however, not limited to ice hockey, and we believe that, in the future, these technologies and systems will play an important role in motion tracking and analysis for a wide range of sports. 

## Figures and Tables

**Table 1:** For the papers discussed in the related work and this paper, we compare the sport/activity, whether activity recognition is used, is a position estimate determined and where the sensors are positioned.

Paper	Sport	Activity Recognition	Positioning Estimate	Sensor Position
[15]	Wheelchair court sports	**✓**	**✗**	On equipment, extra mount on wheelchair
[16]	Handball	**✓**	**✗**	On body, back
[18]	Indoor track cycling	**✓**	**✗**	On equipment, helmet
[19]	Tennis	**✓**	**✓**	On body, UWB on head and IMU on wrist
[20]	Ice hockey	**✓**	**✗**	On body
[21]	Squash, tennis, badminton	**✗**	**✓**	On body, wrist
[22]	Badminton	**✗**	**✓**	On body and on equipment, racket’s grip, wrist and upper arm
[23]	Tennis	**✗**	**✓**	On body, wrist
[24]	Ice hockey	**✗**	**✓**	On and inside equipment (hockey stick)
[10]	Table tennis	**✗**	**✓**	On equipment, front-end of the racket handle
[11]	Baseball	**✗**	**✓**	On body, pelvis and hand
[12]	Basketball	**✗**	**✓**	On body, wrist
[13]	Beach volleyball	**✗**	**✓**	On body, wrist
[14]	Swimming	**✗**	**✓**	On body, legs
**Our paper**	**Ice hockey**	**✓**	**✓**	**Inside equipment (hockey stick) and on body**

**Table 2:** Three different data campaigns were performed to evaluate different parts of the system, before combining them in the fourth and final data campaign.

Measurement Campaign	Technology	Location	Ground Truth	Time (h)
1	IMU	Ice Rink	**✓**	1.2
2	UWB	Controlled lab	**✓**	0.166
3	UWB	Ice Rink	**✗**	0.66
4	IMU + UWB	Ice Rink	only IMU	0.166

**Table 3:** The total available data of the six activities for all players.

Player	Backhand Shots	Slap Shots	Snap Shots	Wrist Shots	Dribbling	Hockey Stance
1 (L)	20	17	20	17	22	14
2 (L)	14	13	12	12	9	13
3 (L)	14	15	14	16	11	9
4 (R)	14	15	15	17	14	6
5 (L)	11	15	15	14	8	7
Total	73	75	76	76	64	49

**Table 4:** Summary of the model structure, the sequence of used layers, the amount of trainable parameters and the output dimensions for each layer.

Layer (Type)	# Parameters	Output Dimension
Input ${}^{1}$	0	150 × 6 × 1
Conv2D (32, 3 × 1), ReLU, kernel_regularizer, bias_regularizer	128	148 × 6 × 32
Maxpool2D (2 × 1), ReLU	0	74 × 6 × 32
Conv2D (16, 3 × 1), ReLU	1552	72 × 6 × 16
Conv2D (16, 3 × 3), ReLU	2320	70 × 4 × 16
Flatten	0	4480
Dense (64), ReLU, kernel_regularizer, bias_regularizer	286,784	64
Dropout 35%	0	64
Dense (16), ReLU	1040	16
Dropout 25%	0	16
Dense (6), Softmax	102	6
Total trainable parameters: 291,926		

${}^{1}$ Input dimension for a 100 Hz sampling frequency.

**Table 5:** Accuracy score for each fold (player).

Player	Accuracy
Player 1 (L)	69%
Player 2 (L)	74%
Player 3 (L)	89%
Player 4 (L)	79%
Player 5 (L)	73%
Player 1 (R)	69%
Player 2 (R)	77%
Player 3 (R)	90%
Player 4 (R)	79%
Player 5 (R)	73%
Total	76%

**Table 6:** The detection and recognition results for each repetition of the continuous movement.

Experiment	# Total Detected Activities	# Correctly Predicted Activities (Total)
Repetition 1	21	21
Repetition 2	29	29
Repetition 3	20	18

**Table 7:** Measurement results to determine the influence of a stick on the UWB signal propagation.

Reference Distance (m)	Free Space (m)	Stick (m)	Difference (m)
1	0.697	0.714	0.017 (2.44%)
2	1.721	1.764	0.043 (2.51%)
5	4.849	4.842	−0.007 (−0.14%)
10	9.768	9.796	0.028 (0.29%)
15	14.764	14.783	0.019 (0.13%)
16	15.754	15.801	0.047 (0.30%)
17	16.673	16.805	0.132 (0.79%)
18	17.772	17.825	0.053 (0.30%)
19	18.767	18.809	0.042 (0.22%)
20	19.766	19.822	0.056 (0.28%)

**Table 8:** Possible ice hockey measurements that can be performed together with their relevance for strategy or single player feedback. The last column indicates whether these are possible with the system discussed in this paper.

Measurement	Input	Relevance		This Paper
		Player	Strategy	
Time spend in the three zones ${}^{1}$	UWB	9	9	**✓**
Team/player positioning	UWB	9	9	**✓**
Power play/penalty kill time ${}^{2}$	UWB	8	8	**✓**
Distance between players	UWB	9	9	**✓**
Total time on ice & shift duration	UWB	9	/	**✓**
Reached skating velocities	UWB	7	/	**✓**
Amount of penalties taken	UWB	7	/	**✓**
Covered distance	UWB	7	/	**✓**
Speed of puck	UWB	6	6	**✗**
Position of puck	UWB	/	8	**✗**
Team puck possession	UWB	/	6	**✗**
Puck position relative to players	UWB	6	7	**✗**
Number of shots	IMU	9	9	**✓**
Face-offs won/lost	IMU + UWB	8	8	**✗**
Detect and recognise shots + location	IMU + UWB	9	9	**✓**
Percentage of shots scored	IMU + UWB	9	9	**✗**
Passes player to teammate	IMU + UWB	/	7	**✗**

${}^{1}$ The ice rink is divided in three zones: attacking, neutral and defensive zone. ${}^{2}$ During a penalty the offending player is sent to the penalty box.

## Data Availability

The data used in this study is available on request from the corresponding author.

## Abbreviations

The following abbreviations are used in this manuscript:

AoA	Angle of Arrival
CNN	Convolutional Neural Network
CSV	Comma Separated Value
IMU	Inertial Measurement Unit
MEMS	Micro-ElectroMechanical Systems
MOCAP	Motion Capturing
ReLU	Rectified Linear Unit
SSH	Secure Shell
TDoA	Time Difference of Arrival
TWR	Two Way Ranging
UWB	Ultra-wideband
